# Model-informed COVID-19 vaccine prioritization strategies by age and serostatus

**DOI:** 10.1126/science.abe6959

**Published:** 2021-01-21

**Authors:** Kate M. Bubar, Kyle Reinholt, Stephen M. Kissler, Marc Lipsitch, Sarah Cobey, Yonatan H. Grad, Daniel B. Larremore

**Affiliations:** 1 Department of Applied Mathematics, University of Colorado Boulder, Boulder, CO 80309, USA.; 2 IQ Biology Program, University of Colorado Boulder, Boulder, CO 80303, USA.; 3 Department of Computer Science, University of Colorado Boulder, Boulder, CO 80309, USA.; 4 Department of Immunology and Infectious Diseases, Harvard T. H. Chan School of Public Health, Boston, MA 02115, USA.; 5 Center for Communicable Disease Dynamics, Harvard T. H. Chan School of Public Health, Boston, MA 02115, USA.; 6 Department of Ecology and Evolution, University of Chicago, Chicago, IL 60637, USA.; 7 BioFrontiers Institute, University of Colorado Boulder, Boulder, CO 80303, USA.

## Abstract

Limited initial supply of severe acute respiratory syndrome coronavirus 2 (SARS-CoV-2) vaccine raises the question of how to prioritize available doses. We used a mathematical model to compare five age-stratified prioritization strategies. A highly effective transmission-blocking vaccine prioritized to adults ages 20 to 49 years minimized cumulative incidence, but mortality and years of life lost were minimized in most scenarios when the vaccine was prioritized to adults greater than 60 years old. Use of individual-level serological tests to redirect doses to seronegative individuals improved the marginal impact of each dose while potentially reducing existing inequities in COVID-19 impact. Although maximum impact prioritization strategies were broadly consistent across countries, transmission rates, vaccination rollout speeds, and estimates of naturally acquired immunity, this framework can be used to compare impacts of prioritization strategies across contexts.

## Vaccine prioritization

There is likely to be high demand for the limited supplies of vaccine against severe acute respiratory syndrome coronavirus 2 (SARS-CoV-2), so how should vaccine distribution be prioritized? Bubar *et al.* modeled across countries how uncertainty about a vaccine's characteristics affects prioritization strategies for reducing deaths and transmission (see the Perspective by Fitzpatrick and Galvani). In the model, vaccine efficacy and its ability to reduce disease and/or block transmission was accounted for in relation to age-related variations in susceptibility, fatality rates, and immune decline. In almost all circumstances, reducing fatalities required distributing the vaccine to those who are most at risk of death, usually persons over 60 years of age and those with comorbidities. If a vaccine is leaky or poorly efficacious in older adults, then priority could be given to younger age groups. To increase the available doses, further priority should be given to seronegative individuals.

*Science*, this issue p. 916; see also p. 890

## Teaser

To minimize mortality, vaccinate seronegative persons most at risk of death: those with comorbidities and those 60+ years of age.

Severe acute respiratory syndrome coronavirus 2 (SARS-CoV-2) has caused a public health and economic crisis worldwide. As of January 2021, there have been more than 85 million cases and 1.8 million deaths reported (*1*). To combat this crisis, a variety of nonpharmaceutical interventions have been implemented, including shelter-in-place orders, limited travel, and remote schooling. Although these efforts are essential to slowing transmission in the short term, long-term solutions—such as vaccines that protect from SARS-CoV-2 infection—remain urgently needed. The benefits of an effective vaccine for individuals and their communities have resulted in widespread demand, so it is critical that decision-making on vaccine distribution is well motivated, particularly in the initial phases when vaccine availability is limited (*2*).

We used a model-informed approach to quantify the impact of COVID-19 vaccine prioritization strategies on cumulative incidence, mortality, and years of life lost. Our approach explicitly addresses variation in three areas that can influence the outcome of vaccine distribution decisions. First, we considered variation in the performance of the vaccine, including its overall efficacy, a hypothetical decrease in efficacy by age, and the vaccine’s ability to block transmission. Second, we considered variation in both susceptibility to infection and the infection fatality rate by age. Third, we considered variation in the population and policy—including the age distribution, age-stratified contact rates, and initial fraction of seropositive individuals by age—and the speed and timing of the vaccine’s rollout relative to transmission. Although the earliest doses of vaccines will be given to front-line health care workers under plans such as those from the COVAX initiative and the U.S. National Academies of Sciences, Engineering, and Medicine (NASEM) recommendations (*3*), our work is focused on informing the prioritization of the doses that follow. On the basis of regulatory approvals and initial vaccine rollout speeds of early 2021, our investigation focuses generally on scenarios with a partially mitigated pandemic [reproduction number (*R*) between 1.1 and 2.0], vaccines with protective efficacy of 90%, and rollout speeds of 0.2% of the population per day.

There are two main approaches to vaccine prioritization: (i) directly vaccinate those at highest risk for severe outcomes and (ii) protect them indirectly by vaccinating those who do the most transmitting. Model-based investigations of the trade-offs between these strategies for influenza vaccination have led to recommendations that children be vaccinated because of their critical role in transmission (*4*, *5*) and have shown that direct protection is superior when reproduction numbers are high but indirect protection is superior when transmission is low (*6*). Similar modeling for COVID-19 vaccination has found that the optimal balance between direct and indirect protection depends on both vaccine efficacy and supply, recommending direct vaccination of older adults for low-efficacy vaccines and for high-efficacy but supply-limited vaccines (*7*). Rather than comparing prioritization strategies, others have compared hypothetical vaccines, showing that even those with lower efficacy for direct protection may be more valuable if they also provide better indirect protection by blocking transmission (*8*). Prioritization of transmission-blocking vaccines can also be dynamically updated on the basis of the current state of the epidemic, shifting prioritization to avoid decreasing marginal returns (*9*). These efforts to prioritize and optimize doses complement other work showing that under different vaccine efficacy and durability of immunity, the economic and health benefits of COVID-19 vaccines will be large in the short and medium terms (*10*). The problem of vaccine prioritization also parallels the more general problem of optimal resource allocation to reduce transmission, such as with masks (*11*).

## Evaluation of vaccine prioritization strategies

We evaluated the impact of vaccine prioritization strategies using an age-stratified SEIR model (susceptible, exposed, infectious, recovered) because age has been shown to be an important correlate of susceptibility (*12*–*14*), seroprevalence (*12*, *15*), severity (*16*–*18*), and mortality (*19*, *20*). This model includes an age-dependent contact matrix, susceptibility to infection, and infection fatality rate (IFR), allowing us to estimate cumulative incidence of SARS-CoV-2 infections, mortality due to infection, and years of life lost (YLL) (supplementary materials, materials and methods) by means of forward simulations of 1 year of disease dynamics. Cumulative incidence, mortality, and YLL were then used as outcomes by which to compare vaccine prioritization strategies. These comparisons may be explored by using accompanying open-source and interactive calculation tools that accompany this study (*21*).

**Fig. 1:**
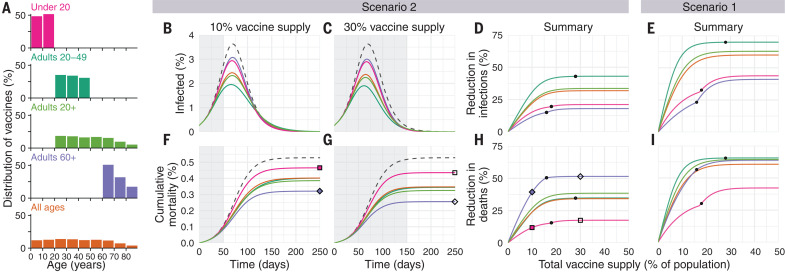
Impacts of vaccine prioritization strategies on mortality and infections. (**A**) Distribution of vaccines for five prioritization strategies: under 20 years, adults 20 to 49 years, adults 20+ years, adults 60+ years, and all ages. (**B**, **C**, **F**, and **G**) Example simulation curves show [(B) and (C)] percentage of the total population infected over time and [(F) and (G)] cumulative mortality for no vaccines (gray dashed lines) and for five different prioritization strategies [colored lines matching (A)], with [(B) and (F)] 10% and [(C) and (G)] 30% vaccine supply. (**D**, **E**, **H**, and **I**) Summary curves show percent reductions in [(D) and (E)] infections and [(H) and (I)] deaths in comparison to an unmitigated outbreak for vaccine supplies between 1 and 50% after 365 days of simulation. Squares and diamonds show how the outputs from single simulations [(F) and (G)] correspond to points in summary curves (H). Gray shading indicates the period during which vaccine is being rolled out at 0.2% of total population per day. Black dots indicate break points at which prioritized demographic groups have been 70% vaccinated, after which vaccines are distributed without prioritization. These simulations assume contact patterns and demographics of the United States (*38*, *53*) and an all-or-nothing, transmission-blocking vaccine with 90% vaccine efficacy and *R*_0_ = 1.5 (scenario 2) and *R*_0_ = 1.15 (scenario 1).

We first examined the impact of five vaccine prioritization strategies for a hypothetical infection- and transmission-blocking vaccine of varying efficacy. The strategies prioritized vaccines to (i) children and teenagers, (ii) adults between ages 20 and 49 years, (iii) adults 20 years or older, (iv) adults 60 years or older, and (v) all individuals (Fig. 1A). In all strategies, once the prioritized population was vaccinated, vaccines were allocated irrespective of age—that is, in proportion to their numbers in the population. To incorporate vaccine hesitancy, at most 70% of any age group was eligible to be vaccinated (*22*).

We measured reductions in cumulative incidence, mortality, and YLL achieved by each strategy, varying the vaccine supply between 1 and 50% of the total population, under two scenarios. In scenario 1, vaccines were administered to 0.2% of the population per day until supply was exhausted, with basic reproduction number (*R*_0_) = 1.15, representing highly mitigated spread during vaccine rollout. In scenario 2, vaccines were administered to 0.2% of the population per day until supply was exhausted, but with *R*_0_ = 1.5, representing substantial viral growth during vaccine rollout (example model outputs are provided in Fig. 1). Results for additional scenarios in which vaccines were administered before transmission began are described in the supplementary materials, supplementary text, corresponding to countries without ongoing community spread such as South Korea and New Zealand. We considered two ways in which vaccine efficacy (*ve*) could be below 100%: an all-or-nothing vaccine, in which the vaccine provides perfect protection to a fraction *ve* of individuals who receive it, or as a leaky vaccine, in which all vaccinated individuals have reduced probability *ve* of infection after vaccination (supplementary materials, materials and methods).

Of the five strategies, direct vaccination of adults older than 60 years of age (60+) always reduced mortality and YLL more than the alternative strategies when transmission was high [*R*_0_ = 1.5, scenario 2, 90% efficacy (Fig. 1); 30 to 100% efficacy (fig. S5)]. For lower transmission (*R*_0_ = 1.15, scenario 1), vaccination of adults aged 20 to 49 years reduced mortality and YLL more than the alternative strategies, but differences between prioritization of adults aged 20 to 49 years, 20+ years, and 60+ years were small for vaccine supplies above 25% (Fig. 1 and fig. S5). Prioritizing adults aged 20 to 49 years minimized cumulative incidence in both scenarios for all vaccine efficacies (Fig. 1 and fig. S5). Prioritizing adults aged 20 to 49 years also minimized cumulative incidence in both scenarios under alternative rollout speeds (0.05 to 1% vaccinated per day) (fig. S6). When rollout speeds were at least 0.3% per day and vaccine supply covered at least 25% of the population, the mortality-minimizing strategy shifted from prioritization of ages 20 to 49 years to adults aged 20+ or 60+ years for scenario 1; when rollout speeds were at least 0.75% per day and covered at least 24% of the population, the mortality-minimizing strategy shifted from prioritization of adults aged 60+ years to adults aged 20+ or 20 to 49 years for scenario 2 (fig. S6). Findings for mortality and YLL were only slightly changed by modeling vaccine efficacy as all or nothing (fig. S5) or leaky (fig. S7).

## Impact of transmission rates, age demographics, and contact structure

**Fig. 2:**
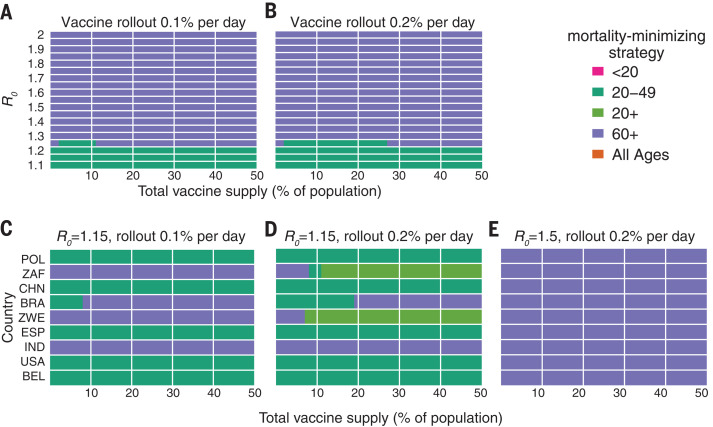
Mortality-minimizing vaccine prioritization strategies across reproductive numbers *R*_0_ and countries. (**A** to **E**) Heatmaps show the prioritization strategies that result in maximum reduction of mortality for varying values of [(A) and (B)] the basic reproductive number *R*_0_ and [(C), (D), and (E)] across nine countries, for vaccine supplies between 1 and 50% of the total population, for an all-or-nothing and transmission-blocking vaccine, 90% vaccine efficacy. [(A) and (B)] Contact patterns and demographics of the United States (*38*, *53*). [(C), (D), and (E)] Contact patterns and demographics of POL, Poland; ZAF, South Africa; CHN, China; BRA, Brazil; ZWE, Zimbabwe; ESP, Spain; IND, India; USA, United States of America; and BEL, Belgium, with *R*_0_ and rollout speeds as indicated.

To evaluate the impact of transmission rates on the strategy that most reduced mortality, we varied the basic reproductive number *R*_0_ from 1.1 to 2.0 when considering a hypothetical infection- and transmission-blocking vaccine with 90% vaccine efficacy. We found that prioritizing adults aged 60+ years remained the best way to reduce mortality and YLL for *R*_0_ ≥ 1.3, but prioritizing adults aged 20 to 49 years was superior for *R*_0_ ≤ 1.2 (Fig. 2, A and B, and fig. S8). Prioritizing adults aged 20 to 49 years minimized infections for all values of *R*_0_ investigated (fig. S8).

To evaluate the impact of transmission rates on the strategy that most reduced mortality, we varied the basic reproductive number *R*_0_ from 1.1 to 2.0 when considering a hypothetical infection- and transmission-blocking vaccine with 90% vaccine efficacy. We found that prioritizing adults aged 60+ years remained the best way to reduce mortality and YLL for *R*_0_ ≥ 1.3, but prioritizing adults aged 20 to 49 years was superior for *R*_0_ ≤ 1.2 (Fig. 2, A and B, and fig. S8). Prioritizing adults aged 20 to 49 years minimized infections for all values of *R*_0_ investigated (fig. S8).

To evaluate the impact of transmission rates on the strategy that most reduced mortality, we varied the basic reproductive number *R*_0_ from 1.1 to 2.0 when considering a hypothetical infection- and transmission-blocking vaccine with 90% vaccine efficacy. We found that prioritizing adults aged 60+ years remained the best way to reduce mortality and YLL for *R*_0_ ≥ 1.3, but prioritizing adults aged 20 to 49 years was superior for *R*_0_ ≤ 1.2 (Fig. 2, A and B, and fig. S8). Prioritizing adults aged 20 to 49 years minimized infections for all values of *R*_0_ investigated (fig. S8).

To determine whether our findings were robust across countries, we analyzed the ranking of prioritization strategies for populations with the age distributions and modeled contact structures of the United States, Belgium, Brazil, China, India, Poland, South Africa, and Spain. Across these countries, direct vaccination of adults aged 60+ years minimized mortality for all levels of vaccine supply when transmission was high (*R*_0_ = 1.5, scenario 2) (Fig. 2E) but in only some cases when transmission was lower (*R*_0_ = 1.15, rollout 0.2% per day, scenario 1) (Fig. 2D). Decreasing rollout speed from 0.2% to 0.1% per day caused prioritization of adults aged 60+ years to be favored in additional scenarios (Fig. 2C). Across countries, vaccination of adults aged 20 to 49 years nearly always minimized infections, and vaccination of adults aged 60+ years nearly always minimized YLL for scenario 2, but no clear ranking of strategies emerged consistently to minimize YLL in scenario 1 (fig. S9).

## Vaccines with imperfect transmission-blocking effects

We also considered whether the rankings of prioritization strategies to minimize mortality would change if a vaccine were to block COVID-19 symptoms and mortality with 90% efficacy but with variable impact on SARS-CoV-2 infection and transmission. We found that direct vaccination of adults aged 60+ years minimized mortality for all vaccine supplies and transmission-blocking effects under scenario 2 and for all vaccine supplies when up to 50% of transmission was blocked in scenario 1 (supplementary text and fig. S10).

## Variation in vaccine efficacy by age

**Fig. 3:**
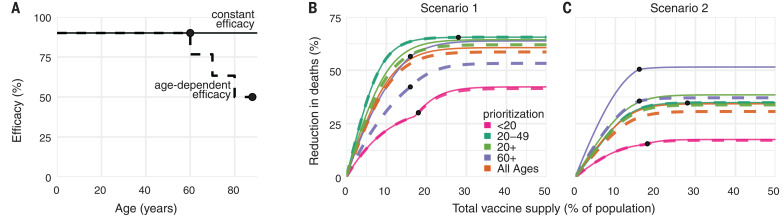
Effects of age-dependent vaccine efficacy on the impacts of prioritization strategies. (**A**) Diagram of hypothetical age-dependent vaccine efficacy shows decrease from 90% baseline efficacy to 50% efficacy among individuals aged 80+ years, beginning at age 60 (dashed line). (**B** and **C**) Percent reduction in deaths in comparison with an unmitigated outbreak for transmission-blocking all-or-nothing vaccines with either constant 90% efficacy for all age groups (solid lines) or age-dependent efficacy shown in (A) (dashed lines), covering scenario 1 [0.2% rollout per day, *R*_0_ = 1.15; (B)] and scenario 2 [0.2% rollout per day, *R*_0_ = 1.5 (C)]. Black dots indicate break points at which prioritized demographic groups have been 70% vaccinated, after which vaccines are distributed without prioritization. Shown are contact patterns and demographics of the United States (*38*, *53*); all-or-nothing and transmission-blocking vaccine.

COVID-19 vaccines may not be equally effective across age groups in preventing infection or disease, a phenomenon known to affect influenza vaccines (*23*–*26*). To understand the impact of age-dependent COVID-19 vaccine efficacy, we incorporated a hypothetical linear decrease from a baseline efficacy of 90% for those younger than 60 years to 50% in those 80 years and older (Fig. 3). As expected, this diminished the benefits of any prioritization strategy that included older adults. For example, strategies that prioritize adults aged 20 to 49 years were unaffected by decreased efficacy among adults aged 60+ years, whereas strategies prioritizing adults aged 60+ years were markedly diminished (Fig. 3). Despite these effects, prioritization of adults aged 60+ years remained superior to the alternative strategies to minimize mortality in scenario 2.

To test whether more substantial age-dependent vaccine effects would change which strategy minimized mortality in scenario 2, we varied the onset age of age-dependent decreases in efficacy, the extent to which it decreased, and the baseline efficacy from which it decreased. We found that as long as the age at which efficacy began to decrease was 70 years or older and vaccine efficacy among adults aged 80+ years was at least 25%, prioritizing adults aged 60+ years remained superior in the majority of parameter combinations. This finding was robust to whether the vaccine was modeled as leaky versus all or nothing, but we observed considerable variation from country to country (fig. S11).

## Incorporation of population seroprevalence and individual serological testing

Because of early indications that naturally acquired antibodies correlate with protection from reinfection (*27*), seroprevalence will affect vaccine prioritization in two ways. First, depending on the magnitude and age distribution of seroprevalence at the time of vaccine distribution, the ranking of strategies could change. Second, distributing vaccines to seropositive individuals would reduce the marginal benefit of vaccination per dose.

To investigate the impact of vaccinating midepidemic while using serology to target the vaccine to seronegative individuals, we included age-stratified seroprevalence estimates in our model by moving the data-specified proportion of seropositive individuals from susceptible to recovered status. We then simulated two approaches to vaccine distribution. In the first, vaccines were distributed according to the five prioritization strategies introduced above, regardless of any individual’s serostatus. In the second, vaccines were distributed with a serological test, so that individuals with a positive serological test would not be vaccinated, allowing their dose to be given to someone else in their age group.

**Fig. 4:**
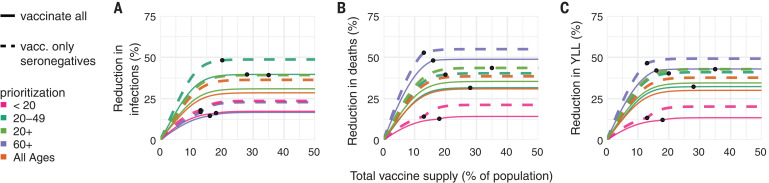
Effects of existing seropositivity on the impacts of prioritization strategies. (**A** to **C**) Percent reductions in (A) infections, (B) deaths, and (C) years of life lost (YLL) for prioritization strategies when existing age-stratified seroprevalence is incorporated [August 2020 estimates for New York City; mean seroprevalence 26.9% (*28*)]. Plots show reductions for scenario 2 (0.2% rollout per day, *R*_0_ = 1.5) when vaccines are given to all individuals (solid lines) or to only seronegatives (dashed lines), inclusive of 96% serotest sensitivity, 99% specificity (*54*), and approximately 3 months of seroreversion (supplementary materials, materials and methods) (*29*). Shown are U.S. contact patterns and demographics (*38*, *53*), all-or-nothing and transmission-blocking vaccine with 90% vaccine efficacy. Lower and higher seroprevalence examples are provided in figs. S12 and S13, respectively.

We included age-stratified seroprevalence estimates from New York City [August 2020; overall seroprevalence 26.9% (*28*)] and demographics and age-contact structure from the United States in evaluations of the previous five prioritization strategies. For this analysis, we focused on scenario 2 (0.2% rollout per day, *R*_0_ = 1.5 inclusive of seropositives) and found that the ranking of strategies to minimize incidence, mortality, and YLL remained unchanged: Prioritizing adults aged 60+ years most reduced mortality, and prioritizing adults aged 20 to 49 years most reduced incidence, regardless of whether vaccination was limited to seronegative individuals (Fig. 4). These rankings were unchanged when we used lower or higher age-stratified seroprevalence estimates to test the consistency of results [Connecticut, July 2020, overall seroprevalence 3.4% (*29*) and synthetic, overall seroprevalence 39.5%] (figs. S12 and S13). Despite lowered sensitivity to detect past exposure due to seroreversion (*30*, *31*), preferentially vaccinating seronegative individuals yielded large additional reductions in cumulative incidence and mortality in locations with higher seroprevalence (Fig. 4 and fig. S13) and modest reductions in locations with low seroprevalence (fig. S12). These results remained unchanged when statistical uncertainty, because of sample size and imperfect test sensitivity and specificity, was incorporated into the model (*32*).

## Discussion

This study demonstrated the use of an age-stratified modeling approach to evaluate and compare vaccine prioritization strategies for SARS-CoV-2. After accounting for country-specific age structure, age-contact structure, infection fatality rates, and seroprevalence, as well as the age-varying efficacy of a hypothetical vaccine, we found that across countries, those aged 60 years and older should be prioritized to minimize deaths, assuming a return to high contact rates and prepandemic behavior during or after vaccine rollout. This recommendation is robust because of the dramatic differences in IFR by age. Our model identified three general regimes in which prioritizing adults aged 20 to 49 years would provide greater mortality benefits than would prioritizing older adults. One such regime was in the presence of substantial transmission-mitigating interventions (*R*_0_ = 1.15) and a vaccine with 80% or higher transmission-blocking effects. A second regime was characterized by substantial transmission-mitigating interventions (*R*_0_ = 1.15) and either rollout speeds of at most 0.2% per day or vaccine supplies of at most 25% of the population. The third regime was characterized by vaccines with very low efficacy in older adults, very high efficacy in younger adults, and declines in efficacy starting at age 59 or 69 years. The advantage of prioritizing all adults or adults aged 20 to 49 versus 60+ years was small under these conditions. Thus, we conclude that for mortality reduction, prioritization of older adults is a robust strategy that will be optimal or close to optimal to minimize mortality for virtually all plausible vaccine characteristics.

By contrast, the ranking of infection-minimizing strategies for midepidemic vaccination led to consistent recommendations to prioritize adults aged 20 to 49 years across efficacy values and countries. For pretransmission vaccination, prioritization shifted toward children and teenagers for leaky vaccine efficacies 50% and below, which is in line with prior work (*7*), as well as for vaccines with weak transmission-blocking properties. Because a vaccine is likely to have properties of both leaky and all-or-nothing models, empirical data on vaccine performance could help resolve this difference in model recommendations, although data are difficult to obtain in practice [for example, (*33*, *34*)].

It is not yet clear whether the first generation of COVID-19 vaccines will be approved everywhere for the elderly or those under 18 years of age (*35*–*37*). Although our conclusions assumed that the vaccine would be approved for all age groups, the evaluation approaches introduced here can be tailored to evaluate a subset of approaches restricted to those within the age groups for which a vaccine is licensed, by using open-source tools such as those that accompany this study. Furthermore, although we considered three possible goals of vaccination—minimizing cumulative incidence, mortality, or YLL—our framework can be adapted to consider goals such as minimizing hospitalizations, intensive care unit occupancy (*7*), or economic costs (*10*).

We demonstrated that there is value in pairing individual-level serological tests with vaccination, even when accounting for the uncertainties in seroprevalence estimates (*32*) and seroreversion (*30*). The marginal gain in effective vaccine supply, relative to no serological testing, must be weighed against the challenges of serological testing before vaccination. Serostatus itself is an imperfect indicator of protection, and the relationship of prior infection, serostatus, and protection may change over time (*10*, *27*, *30*, *31*). Delays in serological test results would impair vaccine distribution, but partial seronegative-targeting effects might be realized if those with past polymerase chain reaction (PCR)–confirmed infections voluntarily deprioritized their own vaccinations.

The best-performing strategies depend on assumptions about the extent of a population’s interactions. We used prepandemic contact matrices (*38*), reflecting the goal of a return to prepandemic routines once a vaccine is available, but more recent estimates of age-stratified contact rates could be valuable in modeling midpandemic scenarios (*39*, *40*). Whether prepandemic or midpandemic contact estimates are representative of contact patterns during vaccine rollout remains unknown and may vary on the basis of numerous social, political, and other factors. The scenarios modeled here did not incorporate explicit nonpharmaceutical interventions, which might persist if vaccination coverage is incomplete, but are implicitly represented in scenario 1 (*R*_0_ = 1.15).

Our study relies on estimates of other epidemiological parameters. In local contexts, these include age-structured seroprevalence and IFR, which vary by population (*19*, *20*, *41*). Globally, key parameters include the degree to which antibodies protect against reinfection or severity of disease and relative infectiousness by age. From vaccine trials, we also need evidence of efficacy in groups vulnerable to severe outcomes, including the elderly. Additionally, it will be critical to measure whether a vaccine that protects against symptomatic disease also blocks infection and transmission of SARS-CoV-2 (*42*).

The role of children during this pandemic has been unclear. Under our assumptions about susceptibility by age, children are not the major drivers of transmission in communities, which is consistent with emerging evidence (*12*). Thus, our results differ from the optimal distribution for influenza vaccines, which prioritize school-age children and adults aged 30 to 39 years (*5*). However, the relative susceptibility and infectiousness of SARS-CoV-2 by age remain uncertain. Although it is unlikely that susceptibility to infection conditional on exposure is constant across age groups (*12*), we ran our model to test the sensitivity of this parameter. Under the scenario of constant susceptibility by age, vaccinating those under 20 years of age has a greater impact on reducing cumulative cases than vaccinating those aged 20 to 49 years (figs. S14 and 15).

Our study is subject to a number of limitations. First, our evaluation strategy focuses on a single country at a time, rather than on between-population allocation (*43*). Second, we only consider variation in disease severity by age. However, other factors correlate with disease outcomes, such as treatment and health care access and comorbidities, which may correlate with factors such as rural versus urban location, socioeconomic status, sex (*44*, *45*), and race and ethnicity (*46*), which are not accounted for in this study. Inclusion of these factors in a model would be possible, but only with statistically sound measurements of their stratified infection risk, contact rates, and disease outcomes. Even in the case of age stratification, contact surveys have typically not surveyed those 80 years and older, yet it is this population that suffers dramatically more severe COVID-19 disease and higher infection fatality rates. We extrapolated contact matrices to those older than 80, but direct measurements would be superior. Last, our study focused on guiding strategy rather than providing more detailed forecasting or estimates (*10*). As such, we have not made detailed parameter fits to time series of cases or deaths but rather have used epidemiologic models to identify robust strategies across a range of transmission scenarios.

Our study also considers variation in disease risk only by age, through age-structured contact matrices and age-specific susceptibility, whereas many discussions around COVID-19 vaccine distribution have thus far focused on prioritizing health care or essential workers (*47*, *48*). Contact rates, and thus infection potential, vary greatly not only by occupation and age but also by living arrangement (such as congregate settings or dormitories), neighborhood and mobility (*49*–*52*), and whether the population has a coordinated and fundamentally effective policy to control the virus. With a better understanding of population structure during the pandemic, and risk factors of COVID-19, these limitations could be addressed. Meanwhile, the robust findings in favor of prioritizing those age groups with the highest IFR to minimize mortality could potentially be extended to prioritize those with comorbidities that predispose them to a high IFR because the strategy of prioritizing the older age groups depends on direct rather than indirect protection.

Vaccine prioritization is not solely a question of science but a question of ethics as well. Hallmarks of the COVID-19 pandemic, as with other global diseases, are inequalities and disparities. Although these modeling efforts focus on age and minimizing incidence and death within a simply structured population, other considerations are crucial, from equity in allocation between countries to disparities in access to health care, including vaccination, that vary by neighborhood. Thus, the model’s simplistic representation of vulnerability (age) should be augmented by better information on the correlates of infection risk and severity. Fair vaccine prioritization should avoid further harming disadvantaged populations. We suggest that after distribution, pairing serological testing with vaccination in the hardest-hit populations is one possible equitable way to extend the benefits of vaccination in settings where vaccination might otherwise not be deemed cost effective.

## Supplementary Materials

science.sciencemag.org/content/371/6532/916/suppl/DC1 Materials and Methods Supplementary Text Figs. S1 to S15 Tables S1 and S2 References (*55*–*57*) MDAR Reproducibility Checklist
